# The evolution of *Brassica napus FLOWERING LOCUST *paralogues in the context of inverted chromosomal duplication blocks

**DOI:** 10.1186/1471-2148-9-271

**Published:** 2009-11-25

**Authors:** Jing Wang, Yan Long, Baoduo Wu, Jia Liu, Congcong Jiang, Lei Shi, Jianwei Zhao, Graham J King, Jinling Meng

**Affiliations:** 1 National Key Laboratory of Crop Genetic Improvement, Huazhong Agricultural University, Wuhan 430070, PR China; 2 Rothamsted Research, Harpenden, Herts, AL5 2JQ, UK

## Abstract

**Background:**

The gene *FLOWERING LOCUS T *(*FT*) and its orthologues play a central role in the integration of flowering signals within *Arabidopsis *and other diverse species. Multiple copies of *FT*, with different *cis*-intronic sequence, exist and appear to operate harmoniously within polyploid crop species such as *Brassica napus *(AACC), a member of the same plant family as *Arabidopsis*.

**Results:**

We have identified six *BnFT *paralogues from the genome of *B. napus *and mapped them to six distinct regions, each of which is homologous to a common ancestral block (E) of *Arabidopsis *chromosome 1. Four of the six regions were present within inverted duplicated regions of chromosomes A7 and C6. The coding sequences of *BnFT *paralogues showed 92-99% identities to each other and 85-87% identity with that of *Arabidopsis*. However, two of the paralogues on chromosomes A2 and C2, *BnA2.FT *and *BnC2.FT*, were found to lack the distinctive CArG box that is located within intron 1 that has been shown in *Arabidopsis *to be the binding site for theFLC protein. Three *BnFT *paralogues (*BnA2.FT*, *BnC6.FT.a *and *BnC6.FT.b*) were associated with two major QTL clusters for flowering time. One of the QTLs encompassing two *BnFT *paralogues (*BnC6.FT.a *and *BnC6.FT.b*) on chromosome C6 was resolved further using near isogenic lines, specific alleles of which were both shown to promote flowering. Association analysis of the three *BnFT *paralogues across 55 cultivars of *B. napus *showed that the alleles detected in the original parents of the mapping population used to detect QTL (NY7 and Tapidor) were ubiquitous amongst spring and winter type cultivars of rapeseed. It was inferred that the ancestral *FT *homologues in *Brassica *evolved from two distinct copies, one of which was duplicated along with inversion of the associated chromosomal segment prior to the divergence of *B. rapa *(AA) and *B. oleracea *(CC). At least ten such inverted duplicated blocks (IDBs) were identified covering a quarter of the whole *B. napus *genome.

**Conclusion:**

Six orthologues of *Arabidopsis FT *were identified and mapped in the genome of *B. napus *which sheds new light on the evolution of paralogues in polyploidy species. The allelic variation of *BnFT *paralogues results in functional differences affecting flowering time between winter and spring type cultivars of oilseed *Brassica*. The prevalent inverted duplicated blocks, two of which were located by four of the six *BnFT *paralogues, contributed to gene duplications and might represent predominant pathway of evolution in *Brassica*.

## Background

Timing of the onset of flowering is an important agronomic trait affecting crop production. To meet the challenges of climate change, and the need to adapt crops to a wider range of growing environments, it is necessary to coordinate flowering within the context of seasonal variations in order to ensure the greatest possibility of pollination, and thus consistently high seed yield. The genetic basis of variation in flowering time is now well understood in *Arabidopsis*. Forward and reverse genetics has allowed identification of genes in the context of environmental and developmental cues that mediate the onset of flowering, and allowed detailed characterization of the photoperiod, vernalization, gibberellin and autonomous pathways. Major integrators of these pathways include *FLOWERING LOCUS T *(*FT*), along with *SUPPRESSOR OF OVEREXPRESSION OF CONSTANS 1 *(*SOC1*) and *LEAFY *(*LFY*) [1-5]. *FT *induces flowering in response to long days and is a direct target of the nuclear protein *CONSTANS *(*CO*) in leaves [6-8]. Two proteins within the vernalization pathway, *FLOWERING LOCUS C *(*FLC*) and *SHORT VEGETATIVE PHASE *(*SVP*), bind to the CArG box within intron 1 and promoter region of *FT*, respectively, to repress its expression [9,10]. *FT *is expressed in the phloem of leaves, with the small protein moving as a long-distance signal to the shoot apical meristem (SAM) where it interacts with *FD*, a bZIP transcription factor, to form a complex of *FT/FD *heterodimer. This then activates the floral meristem identity genes *APETALA 1 *(*AP1*) and *FRUITFUL *(*FUL*) to promote flowering [7,8,11,12].

Although *FT *plays a central and indispensable role to induce flowering, interpreting the exact roles of different *FT *paralogues is difficult, owing to variation in the structure and number of *FT *family members across plant taxa. For example in rice, at least three *FT*-like genes (*OsFTL1*-*OsFTL3*) promote flowering, although there are thirteen such genes within the genome, corresponding to eight in the ancestral grass genome [13-16]. It has been shown that the protein encoded by *Hd3a*, corresponding to *OsFTL2*, is a mobile signal in rice, and moves from the leaf to the SAM to induce flowering [17]. A complex situation also exists in barley, where five *FT*-like genes are found, with *HvFT1 *being highly expressed under long day conditions at the time of transition from vegetative to reproductive growth, and *HvFT2 *and *HvFT4 *expressed later in development. *HvFT3 *is a candidate gene for a major flowering-time QTL, and the expression level of *HvFT5 *is very low in short days, corresponding to a predicted stop codon within the protein at residue 69 [18]. Four *FT*-like cDNAs have been cloned in poplar, of which only *PtFT2 *has been shown to shorten juvenile phase and promote seasonal flowering [19]. Although *Brassica *species share a common ancestor with *Arabidopsis *and diverged at 14.5 to 20.4 MYA [20-22], no *FT *paralogues had previously been identified, apart from 3 RFLP probes from *Arabidopsis FT *having been mapped to the A2 (N2) and C6 (N16) linkage groups of *B. napus *[23].

*B. napus *is a major oil crop in temperate regions worldwide with approximately 52 million tonnes of seed production per year (2007-2008; http://www.worldoil.com/). Results of comparative mapping between the *Arabidopsis *and *Brassica *genomes suggest that numerous regions homologous to the *Arabidopsis *genome are triplicated within diploid species of *Brassica *[24-27]. The *B. rapa *(2n = 20, AA) and *B. oleracea *(2n = 18, CC) genomes are essential conserved intact within the amphidiploid species *B. napus *(2n = 38, AACC), which appears to have been synthesized within the past ten thousand years [24-26,28-31]. Recently, more detailed evidence for the ancestral segmental chromosomal duplications leading to effective triplication in *Brassica *diploids has been obtained from partial genome sequencing and comparative chromosome painting [22,25,32-34]. However, comparative sequence analysis indicates that various mechanisms of genome evolution have contributed to many situations where either more or less than three paralogous genes, corresponding to single orthologues in *Arabidopsis*, are present in the *Brassica *A or C genome [26,28,35-37].

Although there are five homologs of *FT *in *Arabidopsis*, i.e. *TERMINAL FLOWER 1 *(*TFL1*), *TWIN SISTER OF FT *(*TSF*), *ARABIDOPSIS THALIANA CENTRORADIALIS *(*ATC*), *BROTHER OF FT AND TFL1 *(*BFT*), and *MOTHER OF FTAND TFL1 *(*MFT*), their functions are very diverged from *FT*, and only share 56-82% identity at the amino acid sequence level, with *TSF *having the highest similarity [38]. This information allows us to identify *FT *orthologues from the *B. napus *genome unambiguously. We sought to characterize the role that ancestral segmental genome duplication events had played on the number and function of *FT *orthologues within *B. napus*, and to establish how this had contributed to the ability to adapt to contrasting agricultural systems, such as those associated with spring and winter seasonal crop types. This paper presents the results of cloning and characterizing the *BnFT *paralogues, and discusses the evolution of *BnFT *paralogues within *B. napus*.

## Results

### Isolation and genetic mapping of *BnFT *paralogues

**Figure 1:**
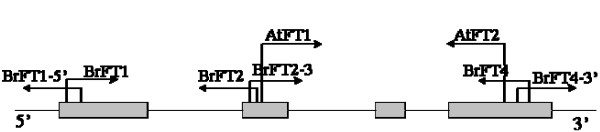
**Distribution of primers used to amplify the *BnFT *fragments, with arrows indicating direction**. The top primer pairs (AtFT1 and AtFT2) were used to amplify the probe for screening *BnFT *paralogues from BAC clones of the JBnB library. Lower primers were used to amplify different regions of *BnFT *paralogues from positive BACs: BrFT1/BrFT2 for exon1-exon2, BrFT2-3/BrFT4 for exon2-exon4; 5' and 3' UTR were amplified with BrFT1-5' and BrFT4-3', respectively, by PCR walking.

Thirty-five potential *FT *orthologue-containing BAC clones were identified by Southern blot screening of the JBnB BAC library, which was developed from the doubled haploid *B. napus *cv. Tapidor [30]. Eleven of these were verified by PCR amplification. These BACs were initially grouped according to intron length (4 groups), and then grouped according to the polymorphism within intron 2 (6 groups) (Additional file 1). Full-length genomic sequences of these six *BnFT *paralogues were isolated from representative BACs by amplification and PCR walking, with primers designed from one copy of *BrFT *(designated as *BrA2.FT*) located in chromosome A2 of *B. rapa *(Figure 1, Table 1).

**Table 1:** Primers designed for isolating and mapping *BnFT *paralogues.

Primer name	Primer sequence
	
For amplifying *BnFT *probe from Tapidor genome	
AtFT1	5'ACCCTCACCTCCGAGAATATCT3'
AtFT2	5'AGCCACTCTCCCTCTGACAAT3'
	
For isolating *BnFT *sequences from positive BACs	
BrFT1	5'AGTTGTAGGAGACGTTCTTGA3'
BrFT2	5'AGATATTCTCGGAGGTGAGGAT3'
BrFT2-3	5'ATGGTGGATCCAGATGTTCCAA3'
BrFT4	5'AGCCACTCTCCCTCTGACAAT3'
BrFT1-5'	5'TTGGCCGTATGTAACCCTTAGATCGAT3'
BrFT4-3'	5'ACAATTCAACACTCGTGAGTTTGCT3'
	
For mapping *BnFT *paralogues	
BnA2.FT-1	5'TCGAAGTGAGTAGGACTAGGT3'
BnA2.FT-2	5'TGCTCTAAGTGACTCCAAAACA3'
BnA7.FTa-1	5'CTCAGGTTCAAAACAAGCCAA 3'
BnA7.FTa-2	5'GATATTCTCGGAGGTGAGGGTT3'
BnA7.FTb-1	5'TGCATAGGAGACATCGAAGTT3'
BnA7.FTb-2	5'AGTTCTATAGGAGTGGACACA3'
BnC2.FT-1	5'ACCCTCACCTCCGAGAATATCT3'
BnC2.FT-2	5'AGCCACTCTCCCTCTGACAAT3'
BnC6.FTa-1	5'AGTAGGACCAGGTTTCCTGT3'
BnC6.FTa-2	5'TGGTATGGTGCATGCATACT3'
BnC6.FTb-1	5'ACGAGAATATCTCCATTGGT3'
BnC6.FTb-2	5'TCCATGTCTAGTTGTGT3'

**Figure 2:**
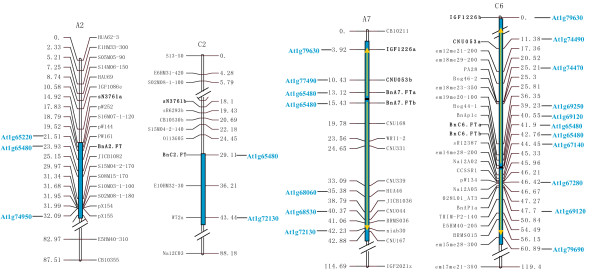
**Genetic mapping of *BnFT *paralogues in the TNDH linkage map**. Loci of *Arabidopsis *are colored light blue to designate homologous markers; Light blue boxes correspond to E segments of *Arabidopsis *chromosome 1. Loci in bold are the markers in common between conserved blocks of the A and C genomes. Large arrows indicate the inverted duplications in A7 and C6 in relation to arrangement of *Arabidopsis *loci.

The six *BnFT *paralogues were mapped using PCR-derived markers to four linkage groups of the TNDH genetic map, two in the A genome and two in the C genome (Figure 2). The *BnFT *paralogues within *B. napus *were named as follows: *BnA2.FT*, *BnA7.FT.a*, *BnA7.FT.b*, *BnC2.FT*, *BnC6.FT.a *and *BnC6.FT.b *according to their mapped chromosomes. *In silico *mapping indicates that all of the genomic regions containing *BnFT *paralogues corresponded to block E in chromosome 1 of *Arabidopsis *[26], although block E was inversely duplicated on linkage groups A7 and C6, where it forms inverted duplication blocks (IDBs). Based on marker identity, the *BnFT *paralogues in the IDBs were close to the junctions of each duplicated block (Figure 2).

**Figure 3:**
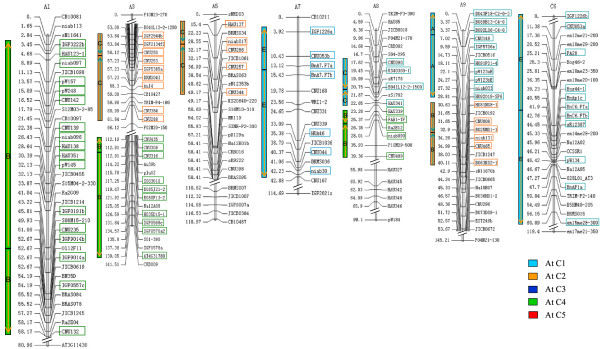
**Detection of IDBs in TNDH linkage map**. The different colored blocks correspond to the different *Arabidopsis *chromosomes (refer to Parkin et al., 2005), with genetic loci labelled according to the *Arabidopsis *homologue, as shown in Additional file 2. IDBs that show conservation of marker content number and marker order between the *Arabidopsis *and *B. napus *genomes are shown to the left of each linkage group. The arrowheads are listed in the order of *Arabidopsis *loci from top to bottom of their respective chromosomes.

According to marker identity, ten IDBs were detected within the TNDH linkage map associated with eight chromosomes (Figure 3, Additional file 2 ). The IDBs covered a quarter of the whole linkage groups of *B. napus *that could align with the genome of *Arabidopsis *(Table 2). Interestingly, blocks located in chromosome 3 and 5 of *Arabidopsis *[26,39] rarely corresponded with the IDBs in the genome of *B. napus*. The majority of IDBs were detected in the A genome and not in the corresponding regions of the C genome, since most markers had been developed from *B. rapa *BACs.

**Table 2:** The coverage of IDBs in regions of the *B. napus *genome which aligned with the *Arabidopsis *genome.

Genome of *B. napus*	Total aligned length of IDB (cM)	Total aligned length of linkage groups (cM)	The ratio of IDB/total aligned genome (%)
A	221.06	837.9	26.38
C	60.89	297.3	20.48
A+C	281.95	1135.2	24.84

### *BnFT *paralogues: candidate genes of QTL for flowering time?

Two *BnFT *paralogues, *BnC6.FT.a *and *BnC6.FT.b*, were mapped previously to a major QTL cluster region for flowering time, which was detected in all winter-cropped environments, with the Tapidor alleles contributing to accelerated flowering [39]. The *BnA2.FT *was newly identified to be located within the confidence interval region of the QTL cluster on chromosome A2 in two winter-cropped environments, with the NY7 allele promoting flowering (Figure 4A). To test whether the *BnC6.FT.a *and *BnC6.FT.b *represented candidate genes of QTL for flowering time, a DH line harbouring the complete C6 QTL cluster in Tapidor was backcrossed to NY7 for four generations, and two near isogenic lines (NILs) with different combinations of introgressed segments on C6, NI-5 and NI-9, were selected. Both NILs flowered much earlier than NY7 (Figure 4B). NI-5, which had a small additional introgressed fragment containing two *BnC6.FT *alleles of Tapidor, significantly flowered earlier than NI-9 which lacked Tapidor alleles (Figure 4B). To further confirm whether the *BnFT *candidates significantly affected flowering time in *B. napus*, *BnC6.FT.a*, *BnC6.FT.b *and *BnA2.FT *were subjected to association analysis with 35 spring and 20 winter cultivars (Additional file 3). The flowering time of the accessions was investigated in vernalization-free conditions, and the genotypes tested with the PCR markers. The NY7 and Tapidor alleles of three *BnFT *paralogues were significantly (P < 0.01) ubiquitous in the spring and winter type cultivars (Table 3). In particular, two paralogues, *BnC6.FT.a *and *BnC6.FT.b*, showed 95% similarity with Tapidor alleles in winter type cultivars (Table 3). The functional differences observed between spring and winter cultivars implied that the expression of *BnFT *candidates was repressively regulated prior to vernalization.

**Table 3:** Association analysis of three *BnFT *paralogues with crop types in cultivars of *B. napus*

	No. winter type cultivar		No. spring type cultivar		
		
Name of paralogues	With Tapidor allele	With NY7 allele	With Tapidor allele	With NY7 allele	χ^2#^
*BnA2.FT*	15	5	4	31	20.08
*BnC6.FT.a*	19	1	4	31	32.91
*BnC6.FT.b*	19	1	5	30	30.69

# χ^2^_0.01, 1 _= 6.63

**Figure 4:**
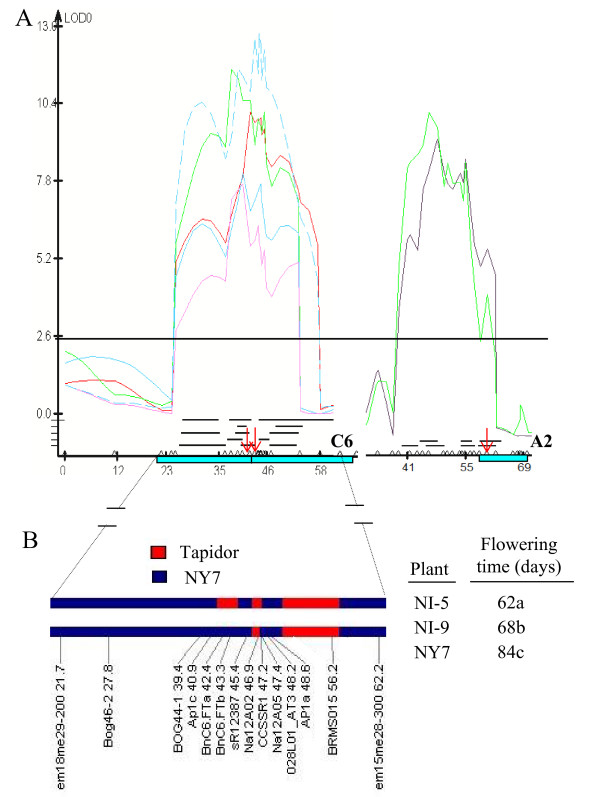
**QTLs of flowering time in A2 and C6 linkage groups of *B. napus***. Light blue boxes represent E segments of *Arabidopsis *chromosome 1. Black lines indicate the confidence interval of flowering time QTL estimated from different environments. (A) A QTL cluster located in C6 (designated as *qcFT.C6*). An inverted duplication corresponding to E segments of *Arabidopsis *chromosome 1 was located in the cluster region. The two red arrowheads indicate the position of *BnC6.FT.a *and *BnC6.FT.b*. QTL detected from the two winter-cropped environments in A2 linkage group and red arrowhead indicates the position of the locus *BnA2.FT*.(B) Two NILs (BC_4_F_4_) with Tapidor introgressions in *qcFT.C6 *region and NY7 as control grown in Gansu (spring-cropped environment). Red boxes represent known homozygous Tapidor introgressions, blue boxes represent homozygous recurrent parent (NY7). An average of 10 individuals per line was analyzed.

### Characterization of *BnFT *paralogues

**Figure 5:**
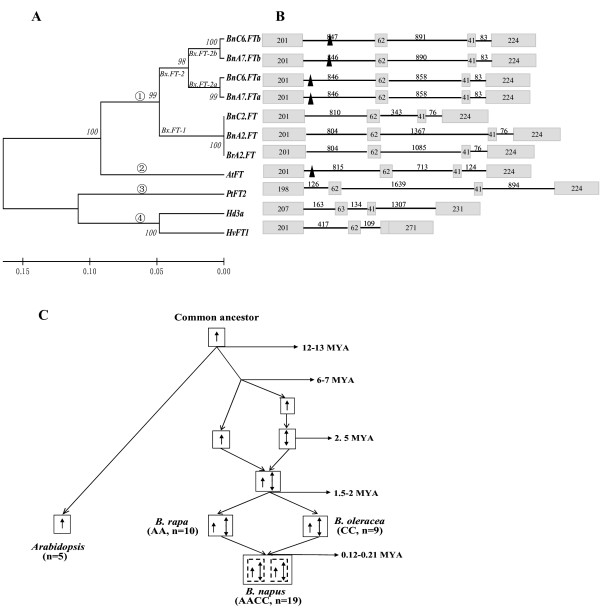
**The phylogenetic relationship of *FT *proteins among *Arabidopsis*, rice, barley, poplar and *B. napus***. (A) Phylogenetic tree of FT amino acid sequences. Major groups are marked 1- 4. (B) Exon/intron structure of *Arabidopsis FT*, rice *Hd3a*, barley *HvFT1*, poplar *PtFT2 *and six *B. napus FT *paralogues. Sizes of exon/intron are in base pairs. Black triangle indicates CArG boxes within intron 1. (C) Chronology of evolutionary events for homologous genes in duplication blocks; Arrow showed the E segments containing *FT *gene.

The canonical organization of *FT *into four exons and three introns as found in *Arabidopsis *(Figure 5B) is well conserved across all the *FT*-like genes identified in rice, poplar and barley genomes. However, some variation of intron size is observed, with the third intron of *HvFT1 *being absent. The number and exon size of all *BnFT *paralogues in *B. napus *were identical to their orthologues in *Arabidopsis*. However, the intron size of all these genes, especially intron 2, was found to differ with that in *Arabidopsis *(Figure 5B). Thus a larger ORF is found for *BnA2.FT *and a smaller ORF for *BnC2.FT*. In both cases a single base deletion is present within intron 1 that disrupts the CArG box, the binding motif for FLC protein to repress *FT *expression prior to vernalization in *Arabidopsis *[10].

Over the coding sequence, the six *BnFT *paralogues had 85-87% identity with *AtFT*, 81-83% with *AtTSF *[GenBank: NM_118156], and 92-99% with each other (Table 4). Interestingly, the paralogues within homeologous regions of the *B. napus *A and C genomes, such as *BnA2.FT *and *BnC2.FT*, showed the highest nucleotide identity (99%), whilst the paralogues present within inverse duplicated regions, such as *BnC6.FT.a *and *BnC6.FT.b*, showed higher identity (97%) than among other pairs of paralogues, such as *BnA2.FT *and *BnC6.FT.a *(92%). The degree of amino acid identity showed the same relationships as the coding sequences (Table 4). The *BnFT *paralogues showed much higher similarity with *AtFT *than with *AtTSF*, indicating that conserved amino acids had been substituted at some sites, although the proteins are likely to perform similar functions.

**Table 4:** Sequence identity among *BnFT *paralogues, *AtFT *and *AtTSF*.

	Identity (%)						
	
Gene	*AtFT*	*BnA2.FT*	*BnA7.FT.a*	*BnA7.FT.b*	*BnC2.FT*	*BnC6.FT.a*	*BnC6.FT.b*
*BnA2.FT*	*87 *85*						
*BnA7.FT.a*	*86 ***81**	*92 ***90**					
*BnA7.FT.b*	*85 ***80**	*92 ***90**	*97 ***94**				
*BnC2.FT*	*87 ***85**	*99 ***99**	*92 ***90**	*92 ***90**			
*BnC6.FT.a*	*86 ***81**	*92 ***89**	*99 ***99**	*97 ***89**	*92 ***89**		
*BnC6.FT.b*	*86 ***81**	*92 ***90**	*97 ***95**	*99 ***99**	*92 ***90**	*97 ***94**	
*AtTSF*	*83 ***82**	*82 ***78**	*81 ***76**	*81 ***77**	*82 ***78**	*81 ***76**	*82 ***77**

*Number in italics indicates coding sequence and number in bold indicates amino acid sequence.

### Phylogenetic and evolutionary analysis of *FT*-like genes across plant taxa

The phylogenetic relationships among *FT*-like genes from *B. napus*, *B. rapa*, *Arabidopsis*, poplar, rice and barley were analyzed, with a phylogenetic tree constructed from the amino acid sequences of these genes. Four groups could be identified: *Hd3a *[GenBank: AB052943] and *HvFT1 *[GenBank: EU331816], *ptFT2 *[GenBank: AY515152], *AtFT *[GenBank: NM_105222] and *Brassica FT *genes (Figure 5A). The classification of species was consistent with established divergence of plant taxa. It was clear that *Brassica FT *genes fell into three groups: *BnA7.FT.b *and *BnC6.FT.b *in group 1, *BnA7.FT.a *and *BnC6.FT.a *in group 2, and *BnA2.FT*, *BnC2.FT *and *BrA2.FT *in group 3 (Figure 5A).

The Ks values between *AtFT *and eight *Brassica FT *genes (from 0.33 to 0.36) (Table 5) suggest a divergence time of 12 to 13 MYA between *Brassica *and *Arabidopsis *(Figure 5C). The Ks values between *FT *genes evolved from *Bx.FT-1 *(Figure 5A) and *Bx.FT-2 *(0.17 to 0.19), *Bx.FT-2a *and *Bx.FT-2b *(0.071 to 0.072) (Table 5) suggest that the first and second duplications may have occurred in *Brassica *~6-7 MYA and 2.5 MYA, respectively. The divergence of A and C genomes (0.041-0.06) may have occurred at 1.5-2 MYA, with natural *B. napus *(0.0033-0.0058) (Table 5) calculated to have emerged approximately 0.12-0.21 MYA (Figure 5C). The order of the evolutionary events calculated by Ks values is consistent with the established phylogenetic relationship of the *FT *genes.

**Table 5:** Ks values estimated for *AtFT *and *BnFT *paralogues within conserved blocks.

	Ks value							
	
Gene	*BnA2.FT*	*BnC2.FT*	*BnA7.FT.a*	*BnC6.FT.a*	*BnA7.FT.b*	*BnC6.FT.b*	*BrA2.FT*	*BoC6.FT.b*
*AtFT*	0.34	0.34	0.33	0.33	0.36	0.35	0.34	0.36
*BnA2.FT*			0.17		0.19		0.0033	
*BnC2.FT*				0.18		0.19	0.06	
*BnA7.FT.a*					0.72			
*BnC6.FT.a*						0.71		
*BnA7.FT.b*								0.041
*BnC6.FT.b*								0.0058

## Discussion

We isolated and mapped six *BnFT *paralogues from *B. napus*. Those regions containing *BnFT *spanned all E segments of *Arabidopsis *chromosome 1, as previously reported in *B. napus *[26,39,40]. Based on marker identity additional IDBs were identified in A7 and C6 [41,42]. However, our primary interest was to identify the *BnFT *paralogues within *B. napus *and to understand the evolutionary processes and functional consequences associated with their duplication.

### Characterization of *BnFT *paralogues

Although the *FT *family members within *Arabidopsis*, *Brassica*, rice, barley and poplar have similar exon/intron structures (apart from barley *HvFT1*), the number of paralogous genes differs. The single *FT *gene in *Arabidopsis *has been ascribed a "florigen" function that responds to long days [6,12]. In contrast, rice, a short day plant, has thirteen *FT*-like genes, of which only the *Hd3a *protein has been shown to have a "florigen" function, with the roles of other paralogues largely unknown [13,14,17]. Five *FT*-like genes are found in barley and play different roles [18]. *B. napus *is an allotetraploid derived from interspecific hybridization of *B. rapa *and *B. oleracea *[43,44] and is more closely related to *Arabidopsis*. The six *BnFT *paralogues exhibit high levels of nucleotide identity to *AtFT *within coding sequences, which initially suggested that all *BnFT *may have a similar function contributing to the induction of flowering. It is of intrinsic and agronomic interest to determine how the presence of six *BnFT *paralogues may cooperate to regulate onset of flowering in *B. napus*. Based on the analysis of the *cis *and coding sequences of the closely related *BnFT *paralogues, it should be possible to dissect the relative timing and contribution of locus-specific paralogues via expression profiles. One may expect that the presence of multiple paralogous copies provides *B. napus *with additional capacity for exquisite tuning of the network of signals that are integrated from the pathways leading to flowering. This tuning will be mediated in different cultivars through differential expression of alleles at each paralogous locus, and such differentiation will be particularly pronounced between winter and spring type cultivars. More complex interactions with the growing environment may also arise from variation in the epigenetic status of the paralogous genes.

Multiple pathways are integrated by *FT *to control flowering [4], with *FT *being the direct target of *CO*, *FLC *and *SVP *proteins in *Arabidopsis *[9,10,45]. In *Brassica *species, several homologues of *Arabidopsis *flowering pathway genes have been identified, such as four *BnCO *copies (*BnCOa1*, *BnCOa9*, *BnCOb1 and BnCOb9*) in *B. napus *[46], five *BoFLC *paralogues (*BoFLC1*, *BoFLC2*, *BoFLC3*, *BoFLC4 *and *BoFLC5*) in *B. oleracea *[47,48], and four *BrFLC *copies (*BrFLC1*, *BrFLC2*, *BrFLC3 *and *BrFLC5*) in *B. rapa *[49]. Based on the evidence from *B. rapa *and *B. oleracea*, between eight or ten *BnFLC *paralogues are anticipated within the whole genome of *B. napus*. It is tempting to speculate that distinct *BnFT *copies may be the targets of one or more *BnCO *and *BnFLC *paralogues in *B. napus*. A characteristic CArG box, which acts as the *FLC *protein binding site, was detected in the first intron of four *BnFT *paralogues. However, it was absent in *BnA2.FT*, *BnC2.FT *and *BrA2.FT *(Figure 5B). These difference in the structural features of the *BnFT *paralogues strongly suggested that they may have undergone functional differentiation and regulatory variation in the context of polyploidy. It has been suggested that redundancy may create subtle fitness advantages that might only be evident in particular stages of the life cycle, or under particular environmental conditions [50]. Force et al. [51] suggest that complementary degenerative mutations in different regulatory elements of duplicated genes can facilitate the preservation of both duplicates, thereby increasing the long-term opportunities for the evolution of new gene functions. Interestingly, the CArG box was also not detected in *PtFT2*, *Hd3a *and *HvFT1 *(Figure 5B) indicating that polyploidy may provide such *FT *orthologues with the opportunity to explore sequence variation that enables more diverse or subtle functionality within the context of more complex regulatory mechanisms and pathways, harmonized within their own genome.

Earlier genetic mapping revealed that three RFLP markers probed with *Arabidopsis FT *mapped to the A2 (N2) and C6 (N16) linkage groups of *B. napus *[23], although this did not account for the full number of expected *BnFT *paralogues, nor the available sequence information. In this study, we established a preliminary allelic relationship between *BnFT *paralogues and flowering time QTL, and extended this interpretation through association mapping analysis. Firstly, the NI-5 that harbored a small introgression fragment containing Tapidor *BnFT *alleles flowered much earlier than NI-9 (Figure 4B). This indicated that Tapidor might possess alleles of *BnFT *with much stronger flower-promoting functional effect than those of NY7. Secondly, association mapping with three *BnFT *candidates indicated that the alleles of NY7 and Tapidor were significantly prevalent in the spring and winter type rapeseeds, respectively (Table 3). The flowering time pathways in *Arabidopsis *are better defined than in *B. napus*, and it is well known that correct flowering time ensures the greatest chance of pollination, higher seed yield and oil content, and therefore reproduction of crop cultivars. Accurate predictive combination of flowering time QTLs, including those associated with a *Brassica *"florigen" function will accelerate the selection of cultivars with appropriate flowering times for different regions, especially where there is variation in latitude and seasonal temperatures associated with winter environments. The different regulatory mechanism and pathway of flowering between winter and spring types are now able to be analyzed further.

### *Brassica FT *genes and genome evolution

Comparative mapping between *Arabidopsis *and *Brassica *species led Lagercrantz et al. [24] to be the first to propose an ancestral segmental triplication affecting the complete *Brassica *genome. Subsequently, this view has been substantiated by compelling evidence from genetic and cytogenetic studies across most of the *Brassiceae *taxa, which indicate this was achieved by a series of distinct duplication events [25,32,33]. However, the detailed mechanisms underlying the process of sequential duplications are not yet well characterized. Based on the sequences of four paralogous *B. rapa *BAC clones and the homologous 124-kb segment of *A. thaliana *chromosome 5, Yang et al. [22] deduced that three paralogous subgenomes of *B. rapa *emerged through duplications 13 to 17 MYA, very soon after the *Arabidopsis *and *Brassica *divergence occurred at 17 to 18 MYA. Using BAC-FISH techniques, three or six copies of the contig have been identified from 18 species of Brassicaceae [25], and the process of ancestral allohexaploidy *Brassica *genome was further revealed as hybridization between genomes of the ancient diploid and tetraploid [27].

Here, we identified six *BnFT *paralogues in *B. napus *and determined that each of the A and C genomes contained three copies, which is consistent with the established view of *Brassica *genome triplication. Moreover, phylogenetic analysis of complete *BnFT *paralogues in *B. napus *allowed us to determine a clear evolutionary relationship between each other. It is apparent that the *Brassica FT *paralogous genes and the unique *Arabidopsis FT *share the same ancestor. The extant *Brassica FT *genes derived from two ancestral lineages (*Bx*), *Bx.FT-1 *and *Bx.FT-2*. Subsequently *Bx.FT-2 *gave rise to two highly identical *FT *copies, *Bx.FT-2a *and *Bx.FT-2b*, and finally *Bx.FT-1 *together with *Bx.FT-2a *and *Bx.FT-2b *formed the final triplication of *BnFT *genes in A and C genomes (Figure 5A). Thus, we propose an evolutionary pathway of *Brassica FT *genes (Figure 5C) which is in good agreement with the model of diploid *Brassica *genome evolution via hexaploidization proposed by Ziolkowski et al. [27]. It is not possible to define the evolutionary divergences accurately based solely upon *FT *family sequence comparison. However, the divergence time calculated by Ks values placed the likely evolutionary events within a distinct order which was in accordance with the established phylogenetic relationship of the *FT *genes.

Interestingly, the two chromosome fragments where *Bx.FT-2a *and *Bx.FT-2b *are located are within IDBs that appear to represent the most recent duplication event, as indicated by the high sequence identity observed between the orthologues. Moreover, the prevalence of IDBs throughout the genome of *B. napus *(Figure 3, Table 2) suggests that IDBs have been a universal and efficient pathway in the evolutionary development of *Brassica*. IDBs with its dosage effect should generate raw genetic materials for the evolution that can be modified subsequently by natural selection just as general DNA duplications do [52], and the occasionally happened pairing between opposite duplicating segments in the meiosis might bring new variations. The homeologous IDBs in different chromosomes would enhance the chance of homeologous reciprocal or nonreciprocal translocations which were already found in several Canadian and Australian cultivars of *B. napus *where IDBs exchanged between A7 and C6 [53,54]. Lyask et al. [25] found that the most frequent chromosome rearrangements involving the At4-b contig are inversions, whilst comparative mapping has indicated that 43% of *Brassica *genomicregions with homeology to *Arabidopsis *chromosomes involved inversions [35]. It has been suggested that rearrangements such as translocations or inversions might reduce or prevent undesirable pairing and recombination between homeologous chromosomes/chromosome regions and lead to reproductive isolation between populations, and eventually contribute to the speciation processes [55]. Our observation of duplication events leading to inverted duplication segments is in accordance with previous reports on inverted chromosome segments duplicated in the C6 (O6) linkage group of *B. oleracea *[41,42]. However, this differs from other reports where only two E segments of *Arabidopsis *chromosome 1 were found in A and C genomes, respectively [40,56].

## Conclusion

The six *BnFT *paralogues have very high identity between their coding sequences, but vary between their corresponding introns. The CArG box within intron 1 was absent from *BnA2.FT *and *BnC2.FT*, which may lead to functional divergence. However, *BnA2.FT *along with two paralogues on chromosome C6, *BnC6.FT.a *and *BnC6.FT.b*, was associated with two major QTL clusters for flowering time indicating that the "florigen" of *B. napus *may be functionally differentiated between winter and spring type cultivars. The *BnFT *paralogues share the same ancestral gene with the single *FT *of *Arabidopsis*, and have evolved via several duplications and divergence resulting from whole genome polyploidization and the formation of inverted duplication blocks. The characterization of the six *BnFT *paralogues in *B. napus *increases our understanding of *Brassica *genome evolutionary pathways involving genome triplication via multi-stage processes.

## Methods

### Plant materials

A *B. napus *doubled haploid [56] population, designated as TNDH and consisting of 202 lines, was generated from an F_1 _resulting from a cross between a Tapidor DH line (hereafter referred to as Tapidor), a European winter cultivar, and Ningyou7 DH (hereafter referred to as NY7), a Chinese semi-winter cultivar [57]. Near isogenic lines (NILs) were developed with NY7 as the recurrent parent and Tapidor as the donor parent. Fifty-five *B. napus *cultivars (in 2006) and two NILs (in 2008) were sown in a field plot located in Gansu Province, one of the spring rapeseed regions in China, with 10 plants maintained for each line. NY7 was grown as a control in both years. The period from sowing the seeds to the appearance of the first flower for each accession was recorded as flowering time. The student's t test (Family wide error rate P < 0.05) was used to test the significance of the variation in flowering time for NILs and NY7. Winter cultivars did not flower under these field conditions.

### BAC library screening and analysis of clones

A 692 bp *FT *genomic DNA sequence of (exon2 to exon4) with 87% of identity to *FT *of *Arabidopsis *for the coding sequence was isolated from Tapidor using primer pairs AtFT-1 and AtFT-2 (Table 1) designed from an mRNA sequence of *FT *(GenBank accession NM_105222). The 692 bp-*FT*-probe was used to screen the JBnB BAC library which was constructed from genomic DNA of Tapidor by Dr. Ian Bancroft, John Innes Centre, UK [30]. Positive BACs were verified with PCR amplification using the AtFT-1/AtFT-2 primer pairs. *BnFT *paralogues were isolated from six BACs using a set of primers (Figure 1, Table 1) designed from a *FT *orthologues (designated as *BrA2.FT*) of *B. rapa *present within the BAC clone KBrB070J11 that had been mapped to chromosome A2 http://brassica.bbsrc.ac.uk/cgi-bin/gbrowse/jic_brassica/. The PCR products were sequenced, and then assembled to form a contig that was compared with the sequence of *BrA2.FT*. A cDNA sequence from *B. oleracea *(GenBank accession EH425279) was designated as *BoC6.FT.b*, due to the 99% identity with *BnC6.FT.b*.

The TNDH linkage map containing 786 markers [58] was used to map the *BnFT *paralogues with the primers designed from the *BnFT *sequences of Tapidor (Table 1). The mapped *BnFT *paralogues were assigned names according to their linkage group, and where two copies were located in the same linkage group, they were distinguished by suffixes with "a" or "b" corresponding to the order of the markers.

### Gene nomenclature

In this paper, we abbreviate the full gene nomenclature for *Brassica *genes as outlined by Ostergaard & King [59], so that *Bna.FT *becomes *BnFT*. In order to distinguish between copies of genes located on specific chromosomes we on occasion indicate this thus: *BnC6.FT.a *is on chromosome C6.

### Sequence analysis

The coding sequences and amino acids of *BnFT *paralogues were predicted using the software "Softberry FGENESH" online service http://linux1.softberry.com/berry.phtml. Multiple alignments of the deduced amino acid sequences of *FT *from *Arabidopsis *and *Brassica *species along with *PtFT2 *(*Populus deltoides*), *Hd3a *(*Oryza sativa*) and *HvFT1 *(*Hordeum vulgare*) were performed using ClustalW http://www.ebi.ac.uk/. Percentage similarities between pairs of *FT *orthologues were calculated using Align http://blast.ncbi.nlm.nih.gov/bl2seq/wblast2.cgi. Phylogenetic relationships were established using MEGA version 4.1 [60] with neighbour-joining based on Kimura's [61] two-parameter model. Putative motifs in intron1 regions were identified using the "Softberry NSITE-PL" online service http://linux1.softberry.com/berry.phtml.

### Phylogenetic and evolutionary sequence analysis

The coding sequences of all the *FT *orthologues were aligned for further analysis. The fraction of synonymous substitutions (Ks) was obtained using K-Estimator version 6.1 [62]. To estimate the timing of evolution for the different duplication events, we used a median Ks value for each orthologous pair between two blocks. Calculations for the dating of the evolutionary events were carried out using a synonymous mutation rate of 1.4 × 10^-8 ^substitutions per synonymous site per year, which was established for the CHALCONE SYNTHASE gene in eudicots [63]. Divergence time (T) was estimated using the equation T = Ks/2 × 1.4 × 10^-8 ^[22].

The blocks associated with inverted duplications within the *B. napus *genome were identified using the TNDH linkage map and confirmed by the colinear array of *Arabidopsis *loci based on marker identity in the linkage groups http://www.arabidopsis.org/wublast/index2.jsp.

## Authors' contributions

JW performed the isolation and mapping of BnFT paralogues, analysis of IDBs, phylogenetic relationship and Brassica FT evolution, and contributed extensively to the writing of the manuscript. YL mapped the QTL for flowering time, compared the whole genome of B. napus to Arabidopsis with TNDH map, and advised JW for the experiment. BW collected the flowering time data in Gansu. JL screened the JBnB BAC library with Tapidor 692 bp-FT-probe. CJ constructed the new version of the TNDH linkage map. JM first conceived of the ideas, guided the data analysis and revised the manuscript. LS and JZ secured funds (2006AA10Z108) and revised the manuscript. GK critically read and improved the manuscript both in terms of academic content and expression of English. All authors read and approved the final manuscript.

## Supplementary Material

Additional file 1 ***BnFT *paralogues information**. The *BnFT *paralogous genes and corresponding BAC clones. Click here for file

Additional file 2 **Marker information within IDBs**. Markers within the IDBs and corresponding orthologous Arabidopsis gene models. Click here for file

Additional file 3 **Information relating to the cultivars of *B. napus***. The phenotype and genotype of spring and winter type *B. napus* grown in a spring environment. Click here for file
