# Supplementary material for: The evolution of Brassica napus FLOWERING LOCUST paralogues in the context of inverted chromosomal duplication blocks
Source: BMC Evol Biol. 2009 Nov 25;9:271. doi: 10.1186/1471-2148-9-271 (PMC2794288; doi:10.1186/1471-2148-9-271)
Supplement: Additional file 1 — BnFT paralogues information. The BnFT paralogous genes and corresponding BAC clones. [file 1471-2148-9-271-S1.PDF]

Group	BAC	Linkage group	Gene	GenBank accession number
1	JBnB045N08	A2	*BnA2.FT*	FJ848913
2	JBnB006F10<br>JBnB183N23	C2	*BnC2.FT*	FJ848914
3	JBnB144I23	A7	*BnA7.FT.a*	FJ848918
4	JBnB032D06<br>JBnB190I10	A7	*BnA7.FT.b*	FJ848916
5	JBnB104L19	C6	*BnC6.FT.a*	FJ848915
6	JBnB054L06<br>JBnB005A21<br>JBnB034G08<br>JBnB003F03	C6	*BnC6.FT.b*	FJ848917
