# Supplementary material for: The evolution of Brassica napus FLOWERING LOCUST paralogues in the context of inverted chromosomal duplication blocks
Source: BMC Evol Biol. 2009 Nov 25;9:271. doi: 10.1186/1471-2148-9-271 (PMC2794288; doi:10.1186/1471-2148-9-271)
Supplement: Additional file 2 — Marker information within IDBs. Markers within the IDBs and corresponding orthologous Arabidopsis gene models. [file 1471-2148-9-271-S2.PDF]

Marker	Linkage group	Arabidopsis Gene locus	Arabidopsis Block
IGF3222b	A1	AT4G39980	4B
HAU123-1	A1	AT4G37860	
niab097	A1	AT4G36180	
pW157	A1	AT4G34890	
pW248	A1	AT4G34500	
CNU142	A1	AT4G33970	
CNU139	A1	AT4G33240	
niab096	A1	AT4G32551	
pW145	A1	AT4G32180	
IGF0191b	A1	AT4G28530	
CNU235	A1	AT4G23650	
IGF9014b	A1	AT4G22740	
IGF9014a	A1	AT4G22740	4B
IGF0557c	A1	AT4G24790	
CNU132	A1	AT4G33970	
IGF2544b	A3	AT2G30250	2C
IGF2134f2	A3	AT2G30700	
CNU250	A3	AT2G32870	
CNU253	A3	AT2G32870	2C
IGF5385a	A3	AT2G33260	
BRMS043	A3	AT2G34480	
Au14	A3	AT2G36910	
CNU384	A3	AT2G44340	
CNU288	A3	AT2G39990	
CNU435	A3	AT4G16820	4B
CNU306	A3	AT4G16460	
CNU316	A3	AT4G16170	
S003O10	A3	AT4G16070	4B
B085J21-2	A3	AT4G19930	
B080F13-2	A3	AT4G23300	
H035D15-1	A3	AT4G26370	
IGF0570a2	A3	AT4G29940	
IGF0568c	A3	AT4G30110	
AT4G31780	A3	AT4G31780	
HAU137	A5	AT2G41870	2C
niab017	A5	AT2G45540	
CNU286	A5	AT2G45330	2C
CNU257	A5	AT2G39380	
CNU344	A5	AT2G35700	
CNU090	A8	AT1G30580	1C
KS40300-1	A8	AT1G32540	
B041L12-2-1500	A8	AT1G33440	

HAU341	A8	AT1G15780	1C
HAU339	A8	AT4G17390	
FAE1-TF	A8	At4G34250	4B
Ra2E12	A8	AT4G32570	
niab090	A8	AT4G27090	4B
CNU489	A8	AT4G14615	
B043F18-C2-0-3	A9	AT1G04450	1A
B089B13-C4-0	A9	AT1G03080	
B092L06-C4-0	A9	AT1G02260	
CNU148	A9	AT1G04440	1A
IGF5706e	A9	AT1G05205	
H091P21-4	A9	AT1G09570	
pW123aH	A9	AT1G14150	
niab033	A9	AT1G17680	
H092O19-SP6	A9	AT1G17980	
H081N08-1	A9	AT2G20960	2B
CNU008	A9	AT2G21240	
B025M01-1	A9	AT2G21710	
niab131	A9	AT2G21520	2B
CNU465	A9	AT2G21385	
B063K02-1	A9	AT2G21320	
