# Supplementary material for: The evolution of Brassica napus FLOWERING LOCUST paralogues in the context of inverted chromosomal duplication blocks
Source: BMC Evol Biol. 2009 Nov 25;9:271. doi: 10.1186/1471-2148-9-271 (PMC2794288; doi:10.1186/1471-2148-9-271)
Supplement: Additional file 3 — Information relating to the cultivars of B. napus. The phenotype and genotype of spring and winter type B. napus grown in a spring environment. [file 1471-2148-9-271-S3.PDF]

Spring cultivar	Flowering time (day)	Genotype			Winter cultivar	Flowering time (day)	Genotype		
		BnA2.FT	*BnC6.FT.a*	*BnC6.FT.b*			*BnA2.FT*	*BnC6.FT.a*	*BnC6.FT.b*
Altex	69	N	T	T	Apache	None bloom	N	T	T
Alto	65	N	T	T	Bakow	None bloom	N	T	T
Apomix	65	N	N	N	Bienvenu	None bloom	T	T	T
ChuanYou11	67	T	N	N	Bolko	None bloom	T	T	T
D.ARoll	72	N	T	T	Brutor	None bloom	T	T	T
Dac-chosen	67	N	N	N	Doral	None bloom	N	N	N
Erra	69	N	N	N	Casino	None bloom	T	T	T
Fuyou2	67	N	N	N	Ceres	None bloom	T	T	T
Ganyou2	67	N	N	N	Coma	None bloom	N	T	T
Ganyou5	67	N	N	N	Huayou9	None bloom	T	T	T
Ganyou3	69	N	N	N	JeT-NeVT	None bloom	N	T	T
Huayou11	68	N	N	N	Jupiter	None bloom	T	T	T
Huayou13	72	N	N	N	Liradouna	None bloom	T	T	T
Huayou14	71	N	N	N	Lisritta	None bloom	T	T	T
Huayou2	67	T	N	N	Literavo	None bloom	T	T	T
Huayou4	63	N	N	N	Matador	None bloom	T	T	T
Huayou6	67	T	N	N	NESTOR	None bloom	T	T	T
Huayou10	69	N	N	N	Panter	None bloom	T	T	T
Jiayou1	76	N	N	N	Piadem	None bloom	T	T	T
Jiayou3	75	T	N	N	Quinta	None bloom	T	T	T
Lirason	74	N	N	N	Tapidor	None bloom	T	T	T
Major	86	N	N	N					
Mamoo	69	N	T	T					
Nilla	82	N	N	T					
Xiangnongyou2	70	N	N	N					
Xiangnongyou3	67	N	N	N					
Xiangyou13	70	N	N	N					
Youyan2	64	N	N	N					
Huashuang3	75	N	N	N					
Xiangyou13	67	N	N	N					
Qingyou2	65	N	N	N					
Huayou5	69	N	N	N					
Huayou6	61	N	N	N					
Suyou3	62	N	N	N					
Fuyou1	66	N	N	N					
NY7	77	N	N	N					
